# Microevolution of *Pseudomonas aeruginosa* in cystic fibrosis lungs

**DOI:** 10.1186/2194-7791-1-S1-A28

**Published:** 2014-09-11

**Authors:** Nina Cramer, Jens Klockgether, Colin F Davenport, Burkhard Tümmler

**Affiliations:** Klinik für Pädiatrische Pneumologie, Allergologie und Neonatologie, Medizinische Hochschule Hannover, Germany

The chronic airway infections with *Pseudomonas aeruginosa* determine disease outcome in most individuals with cystic fibrosis (CF). We have collected serial *P. aeruginosa* isolates in half year intervals from all 35 CF patients at our local CF clinic who became colonized with *P. aeruginosa* in the 1980s. The microevolution of *P. aeruginosa* in CF lungs was investigated in the six patients with the mildest and most severe course of their chronic *P. aeruginosa* infection.

Serial isolates were genotyped with a customized microarray. Isolates of the initially colonizing clone were then subjected to whole genome sequencing by SOLiD5500 technology. Nucleotide variations compared to the PA14 genome were extracted, filtered, annotated and used for the reconstruction of clades. The 250 sequenced bacterial isolates were characterized in mutation rates, morphology, motility and secretion of virulence effectors.

Exopolysaccharide biosynthesis, antimicrobial resistance and global regulators of lifestyle and metabolism are the most common functional categories whose genes were hit by mutations in the CF lungs. Microevolution was not uniform. The *P. aeruginosa* clone inhabiting severely affected lungs repetitively generated descendants with stop mutations or drastic amino acid changes in key genes of lifestyle, but these loss-of-function mutants were not recovered at later time points. In contrast, *P. aeruginosa* clones predominantly acquired benign amino acid substitutions in patients who maintained a normal function of their chronically colonized lungs for up to 30 years.

Modes of microevolution of *P. aeruginosa* in CF lungs are associated with the severity of the chronic lung infection.

Supported by DFG (SFB900) and Christiane Herzog Stiftung.

